# A pilot study of toceranib/vinblastine therapy for canine transitional cell carcinoma

**DOI:** 10.1186/s12917-016-0882-6

**Published:** 2016-11-17

**Authors:** Sarah B. Rippy, Heather L. Gardner, Sandra M. Nguyen, Emma E. Warry, Roberta A. Portela, William Tod Drost, Eric T. Hostnik, Eric M. Green, Dennis J. Chew, Juan Peng, Cheryl A. London

**Affiliations:** 1 University of Missouri, Columbia, MO USA; 2 Departments of Veterinary Clinical and Biosciences, The Ohio State University, 454 Veterinary Medical Academic Building, 1925 Coffey Rd., Columbus, OH 43210 USA; 3 Premier Veterinary Group of Chicago, Chicago, IL USA; 4 Department of Biostatistics, The Ohio State University, Columbus, OH USA

**Keywords:** Transitional cell carcinoma, Computed tomography, Ultrasound, Toceranib, Vinblastine

## Abstract

**Background:**

Effective therapies for transitional cell carcinoma (TCC) are limited, with objective response rates to most chemotherapeutic regimens below 20%. The purpose of this study was to investigate the biologic activity of combined toceranib phosphate and vinblastine chemotherapy for treatment of TCC. A secondary objective was to compare the utility of Computed Tomography (CT) and abdominal ultrasound (AUS) in tumor response assessments.

**Results:**

Dogs with TCC received vinblastine at 1.6 mg/m2 every 2 weeks and toceranib at 2.5–2.75 mg/kg on Monday/Wednesday/Friday. Tumor monitoring was achieved through CT and AUS. Five patients completed the 16-week study. Based on AUS assessments, 3 dogs experienced biologic response to therapy including partial responses (PR, *n* = 2) and stable disease (SD, *n* = 1). Based on CT, 5 dogs experienced a biologic response (*n* = 2 PR, *n* = 3 SD). Both imaging modalities (ultrasound and CT) were found to provide repeatable measurements between operators, however agreement between operator measurements was greater when CT images were used to assess tumor size.

**Conclusions:**

The combination of toceranib and vinblastine did not result in improved response rates. While agreement in tumor volume assessments between both AUS and CT were excellent between operators, this did not extend to assessment of tumor response. The higher rate of concordance between operators when assessing response to treatment with CT suggests that CT should be considered for future clinical trials involving canine bladder TCC to improve the accuracy and repeatability of tumor measurement. The data suggest that response to therapy as assessed by AUS or CT do not predict duration of clinical response.

## Background

Transitional cell carcinoma (TCC) of the urinary bladder is the most common cancer of the canine bladder, and accounts for 1–2% of all cancers diagnosed in dogs [1–4]. Evidence suggests that certain breeds are at higher risk for developing this disease including the Scottish Terrier, West Highland White Terrier, Beagles, and Shetland Sheep Dogs [5, 6]. Other reported risk factors for the development of TCC in dogs include female sex, obesity and exposure to herbicides [5, 7, 8]. TCC is a frustrating disease for both pet owners and clinicians due to a lack of effective therapies. These tumors are often not amenable to surgical excision as they typically involve the trigone of the bladder and/or occur multifocally throughout the bladder secondary to intravesicle seeding. When surgical removal can be performed it is usually palliative, as local recurrence and distant tumor relapse rates are high with median survival times of only 3–10 months [6, 9, 10]. Aggressive surgical techniques, such as bladder replacement, have been attempted with very limited success. Cystectomy with ureterocolonic anastomosis is associated with severe complications including metabolic acidosis, uremia, pyelonephritis and resulting in survival times of less than 5 months [11]. Urethral stenting in dogs with TCC affecting the urethra/trigone can temporarily relieve tumor related obstruction, although chronic bacterial cystitis and incontinence are significant complications and in many cases, survival times post stenting are less than 3 months [12–14]. Transurethral resection in 6 dogs resulted in urethral perforation in 2 cases and tumor seeding in another 2; furthermore, this procedure cannot be performed in female dogs due to the high risk of fatal complications [15].

Available medical therapies for canine TCC are similarly disappointing with respect to outcome. The COX1/COX2 inhibitor piroxicam has been used for over 20 years to treat canine TCC. While clinical symptoms often improve following piroxicam administration, the objective response rate and survival time are both low (18% and 6 months, respectively) [16, 17]. Several different chemotherapy agents have been used to treat canine TCC including carboplatin, cisplatin, doxorubicin, cyclophosphamide and intravesicle thiotepa. None of these drugs result in objective response rates greater than 10–15% [3, 4]. Piroxicam has been co-administered with carboplatin resulting in no significant improvements in survival [18]. Moreover, while the combination of cisplatin and piroxicam did improve the objective response rate in affected dogs to 71%, fatal nephrotoxicity occurred in several patients making this therapeutic combination unsuitable [19]. Lastly, piroxicam has been combined with the chemotherapeutic mitoxantrone resulting in an objective response rate of approximately 35% [20]. This drug combination is often used to treat TCC although the reported median survival time is only 291 days from the date of diagnosis. More recently, a clinical trial directly compared the use of carboplatin/piroxicam versus mitoxantrone/piroxicam therapy for the treatment of canine TCC [21]. There was no difference in the objective response rate (13 vs 8%) or progression free interval (73 vs 106 days) between the treatment groups. Vinblastine has been used as a single agent to treat canine TCC resulting in a 36% partial response rate, although these were relatively short lived as the progression free survival was only 4 months, with an overall survival time of approximately 5 months [22]. Lastly, the use of metronomic chlorambucil has also showed some clinical efficacy with a reported biological response rate of 68%, although nearly all of these consisted of stable disease [23].

Radiation therapy has also been utilized to treat TCC in dogs, however its use has been limited by lack of durable response times and the development of late complications. In a limited number of cases radiation has been used as the sole treatment modality, with reported median survival times of 4–16 months [4, 6, 24]. Reported outcomes in canine TCC when adjuvant radiation therapy is incorporated into the treatment regimen are inconsistent. When radiation was combined with mitoxantrone and piroxicam in one study, the reported response rate of 22% was not significantly different compared to the use of mitoxantrone and piroxicam alone [25]. More recently, the use of image guided intensity modulated radiation (IG/IMRT) with or without adjunctive chemotherapy in one study reported a median survival time of 614 days [26]. In addition, adjuvant low-dose palliative radiation therapy resulted in a 61% objective response rate, with 100% of dogs experiencing clinical benefit (CR, PR, SD) within 6 weeks of radiation therapy [27]. While in vitro evaluation of α/β ratios in TCC suggest that the cells will respond best to larger fraction size [28], coarsely fractionated protocols are associated with the development of late treatment related complications (39–56%), including urinary incontinence, cystitis, stranguria, colonic/ureteral strictures, colonic perforation and bladder fibrosis [4, 6, 24]. Recently described fractionated protocols report a lower incidence of late effects, with 0–19% of patients developing late dose-limiting side effects [27, 29].

Distension of the urinary bladder is variable over time, making accurate monitoring of tumor size difficult. Inaccurate assessment of tumor size can lead to altering a treatment regimen prior to true progressive disease or long after definitive progression has occurred. Measurements are typically performed with ultrasound, despite the fact that computed tomography (CT) and magnetic resonance imaging (MRI) are the preferred monitoring modality in humans [30]. The veterinary literature has previously evaluated traditional two dimensional ultrasound and found high variability in measurements obtained between operators and between measurements taken by a single operator when a patient had variable bladder distention [31]. A previous study investigated the correlation between measurements taken by two dimensional ultrasound, three dimensional ultrasound, and CT. The findings indicate that three-dimensional ultrasound correlates more closely with CT. However, the study did not evaluate the variability between measurements taken by different operators with each modality [32]. Importantly, ultrasound is not considered an acceptable imaging method for measuring tumors in human oncology due to inherent intra- and inter-operator variables as well as the inability to precisely reproduce images in the same plane over successive assessments [33].

Toceranib phosphate (Palladia) is a multi-targeted receptor tyrosine kinase inhibitor that disrupts the function of several members of the split kinase receptor tyrosine kinase (RTK) family including VEGFR, PDGFR, KIT, and Flt-3, among others [34]. Toceranib demonstrated single agent activity against a variety of tumor types in a phase 1 study in dogs with cancer including several carcinomas [34]. In this clinical trial, 3 of 4 dogs with TCC of the bladder treated with toceranib alone had stable disease for 10 weeks or greater. Data generated from experimental models supports the notion that inhibitors of VEGFR and PDGFR may synergize with chemotherapeutics in the treatment of urothelial carcinomas [35, 36]. As such, the purpose of this clinical trial was to provide a preliminary assessment of the biologic activity of combined toceranib and vinblastine therapy in dogs with TCC confined to the urinary bladder, to evaluate the repeatability of AUS and CT measurements for TCC between different operators, and to compare the utility of AUS and CT for assessing response to therapy.

## Methods

### Eligibility

This clinical trial was approved by the Clinical Research and Advising Committee at the College of Veterinary Medicine and Institutional Animal Care and Use Committee (IACUC) at Ohio State University. Dogs were eligible if they had a TCC of the urinary bladder that measured more than 1 cm in diameter on baseline ultrasound without obvious evidence of urethral involvement. Cytologic confirmation of a diagnosis from either a urine cytospin or traumatic catheterization was required for study entry. Prior to enrollment dogs underwent a series of tests including complete blood count (CBC), biochemistry profile, urinalysis, abdominal ultrasound, and thoracic radiographs. Dogs with distant metastatic disease on diagnostic imaging were not eligible for enrollment, however locoregional lymph node metastasis was permitted. Additional eligibility criteria included age of at least 1 year, ECOG performance score of 0–1, adequate organ function as indicated by routine bloodwork (e.g., liver transaminases <3x the upper limit of normal (ULN), creatinine <1.5x ULN), and no evidence of any serious systemic disorder (e.g., cardiac disease) considered incompatible with the study.

### Drug product and concomitant medication

Toceranib phosphate was provided by Zoetis (Madison, NJ) in 10, 15, and 50 mg size tablets. Concomitant medications considered acceptable for use to prevent and/or treat drug related toxicities included famotidine, omeprazole, metronidazole, loperamide, metoclopramide, ondansetron, maropitant, tramadol, carprofen, meloxicam and/or piroxicam at the discretion of the attending clinician.

### Study design

A total of ten dogs were enrolled in this pilot study. Dogs were administered vinblastine every 2 weeks and toceranib on a Monday/Wednesday/Friday basis. The dose for vinblastine was 1.6 mg/m^2^ given IV every 2 weeks based on a previously published study combining toceranib with vinblastine [37]. The starting dose for toceranib was 2.75 mg/kg with dose de-escalation down to 2.25 mg/kg permitted in the face of adverse events. Dogs already receiving a non-steroidal anti-inflammatory drugs (NSAIDs) and tolerating them without any gastrointestinal side effects continued to receive therapy, however dose frequency was reduced by administration on either Tuesday, Thursday, Saturday, and Sunday or Tuesday, Thursday, Saturday only. Further reduction or discontinuation was clinician dependent and done according to clinical signs. All patients received omeprazole at approximately 1 mg/kg PO q24, with the dose rounded to accommodate available capsule or tablet sizes (10 mg capsules and 20 mg tablets). Dogs were assessed at week 1, then every 2 weeks thereafter for a total of 16 weeks. This included physical examination, patient weight, adverse event assessment, CBC, hemoccult, serum biochemistry profile and urinalysis. A UPC was evaluated as indicated by proteinuria in the absence of pyuria, bacturia, or hematuria.

An abdominal ultrasound was performed prior to treatment then at weeks 4, 8, 12 and 16 of study. A CT scan was performed at baseline and at weeks 8 and 16 of study. Only measurements taken at baseline, week 8, and week 16 were included in the statistical assessment. General anesthesia was used during all measurements taken at baseline, week 8, and week 16. This allowed for the placement of a urinary catheter to ensure a stable bladder volume at these time points. Unscheduled visits were conducted at interim time points as needed. Dogs were evaluated for adverse events (AEs) at every study visit. AEs were defined and graded according to the published VCOG-CTCAE criteria [38]. Disease progression or signs and symptoms definitely related to disease were not considered AEs.

### Tumor measurements: ultrasound

Baseline ultrasound measurements were obtained under anesthesia. Following placement of a urinary Foley catheter, the urinary bladder was emptied and then 2 ml/kg of sterile 0.9% saline was instilled into the urinary bladder. The urinary catheter was withdrawn into the proximal urethra. All sonographic exams were performed with the patient positioned in dorsal recumbency using a 3–12 MHz microconvex transducer (Toshiba Aplio 500, Toshiba America Medical Systems, Tustin, CA 92780). The frequency and depth of imaging were determined by the size of the urinary bladder. Neither harmonics nor image compounding were used. The greatest cranial-caudal axis of the urinary bladder was determined by locating the neck of the urinary bladder and finding the scan plane with the longest urinary bladder dimension. The plane was marked on the patient’s skin using a pen. While maintaining the transducer parallel to the marked sagittal plane, the image with the greatest tumor length was determined. The length of the tumor was measured using the ultrasound machine calipers to the nearest tenth of a centimeter. To measure the maximum tumor height and width, the transducer was oriented 90° to the sagittal plane of the urinary bladder. Maintaining the transverse plane, the tumor was imaged and the maximum width and maximum height of the tumor was recorded. The left and right medial iliac lymph nodes were measured in longitudinal and transverse planes. For each case, two observers independently located the tumor within the urinary bladder and made measurements. One observer (DMA) made images at all 24 time points. One person (WTD) was the second observer on 21 time points and a third observer (EMG) performed measurements on the remaining 3 time points. All observers had at least 16 years of veterinary ultrasound experience.

### Tumor measurements: computerized tomography (CT)

The CT imaging was acquired following completion of the ultrasound examination. Dogs were placed in ventral recumbency and scout images were acquired (GE Light Speed, Milwaukee, WI). For the actual study, 1.25 mm helical images were acquired from the diaphragm through the distal aspect of the urethra. Three separate series were performed as follows: 1. Empty urinary bladder; 2. Infuse 2 ml/kg of 0.9% sterile saline into the urinary bladder; 3. Following intravenous administration of 2 mL/kg of iohexol 240 (Omnipaque, GE Healthcare, Princeton, NJ 08540). Transverse images were reconstructed into sagittal and dorsal plane images.

Measurements were made on post-contrast CT images. The dorsal plane images were initially reviewed to evaluate the position of the urinary bladder relative to the sagittal plane (long axis) of the patient. If the urinary bladder was parallel to the sagittal plane of the patient, the maximum length of the tumor was measured using dorsal or sagittal plane images. The maximum width of the tumor was measured using dorsal or transverse plane images. The maximum height of the tumor was measured using sagittal or transverse plane images. Measurements were made using a DICOM viewer (eFilm Workstation 3.2, Merge Healthcare, Chicago, IL 60654). If the sagittal plane of the urinary bladder was not parallel to the sagittal plane of the patient, orthogonal multi-planar reformats of the urinary bladder were made using the DICOM viewer using the sagittal plane of the urinary bladder as a point of reference. Length, width and height measurements were of the tumor were made using the multi-planar reformatted images to the nearest tenth of a millimeter. Two observers (WTD, ETH) independently made measurements of each tumor at each time point.

### Response assessment

Evaluations of tumor volume were made according to the calculation V = π/6 (L*W*H) which uses the longest diameter in three dimensions to calculate volume [39]. This formula assumes that the tumor will have a roughly ellipsoid shape and has been used to calculate tumor volume from ultrasound images, CT images, and MRI images in both human and veterinary medicine. The volume calculation was applied to the measurements from the independent tumor evaluations in each modality, and then the percent difference between the results were calculated for each modality. Final assessment of the tumor volume at that time point was made using the mean value of the 3 longest diameter measurements obtained in each modality.

### Statistical analysis

The reliability testing for repeated measures on tumor size (volume) was performed using intra-class correlation (ICC) for ultrasound and CT scan, respectively. The ICC is a measurement of variation between the values given to each subject by ultrasound or CT scan, with a value of 1 denoting perfect reliability. Averaged values were calculated across the two repeated measurements at each visit for CT scan. These values were compared with the averaged values at each visit from ultrasound using concordance correlation coefficient (CCC) and Bland-Altman plots for longitudinal repeated measurements. The CCC measures how closely the CT scan measurements adhere to a 45° line (i.e., a line of perfect agreement) when plotted against the Ultrasound values, with a value of 1 denoting perfect agreement. Each analysis required methods to account for within-subject correlations due to repeated measures on the same subject across visits. Limits of agreement (bias ± 1.96 SD) for the Bland-Altman plot were calculated using Bland and Altman’s formula for repeated measures data [40] while CCC and their confidence intervals were calculated using Carrasco, King, and Chinchilli’s method for repeated measures data [41]. All measurements were included in a mixed effect model to test the technician effect and instrument effect on the change of tumor size across visits. A subject-specific random intercept was used to account for within-subject correlation due to the repeated measures. The Kenward-Roger adjustment to the degrees of freedom was used to control Type I error rates [42]. Tumor size values were natural log transformed to better approximate normality of residuals. Kappa statistics and bootstrap confidence intervals were computed to access the agreement between observers and between instruments based on the clinical outcome defined on change of tumor size. A two-sided significance level of α = 0.05 was used for all tests. All analyses were carried out in SAS version 9.4 (SAS institute, Cary, NC).

## Results

### Demographics

Ten dogs were enrolled in the study between 2011 and 2014. The median age was 11.5 (mean 11.5, range 9–14) years with a median weight of 13.6 kg (mean 17.1, range 8.1–29.2). Of those patients, eight were female and two were male. Five patients were mixed breed dogs, four were statistically over-represented breeds (2 West Highland White Terriers, 1 Shetland Sheep Dog, and 1 Scottish Terrier), and 1 was a Greyhound. Two patients had enlarged regional lymph nodes with the potential for metastasis, though disease spread was not confirmed by FNA due to the risk of seeding the peritoneum. The remaining eight had disease confined within the bladder (T2, N0, M0). Nine patients were on NSAIDs prior to starting the trial, and all dogs continued to receive them at a reduced dose intensity (q48 h) while on study.

### Reliability and concordance of tumor assessments

**Fig. 1:**
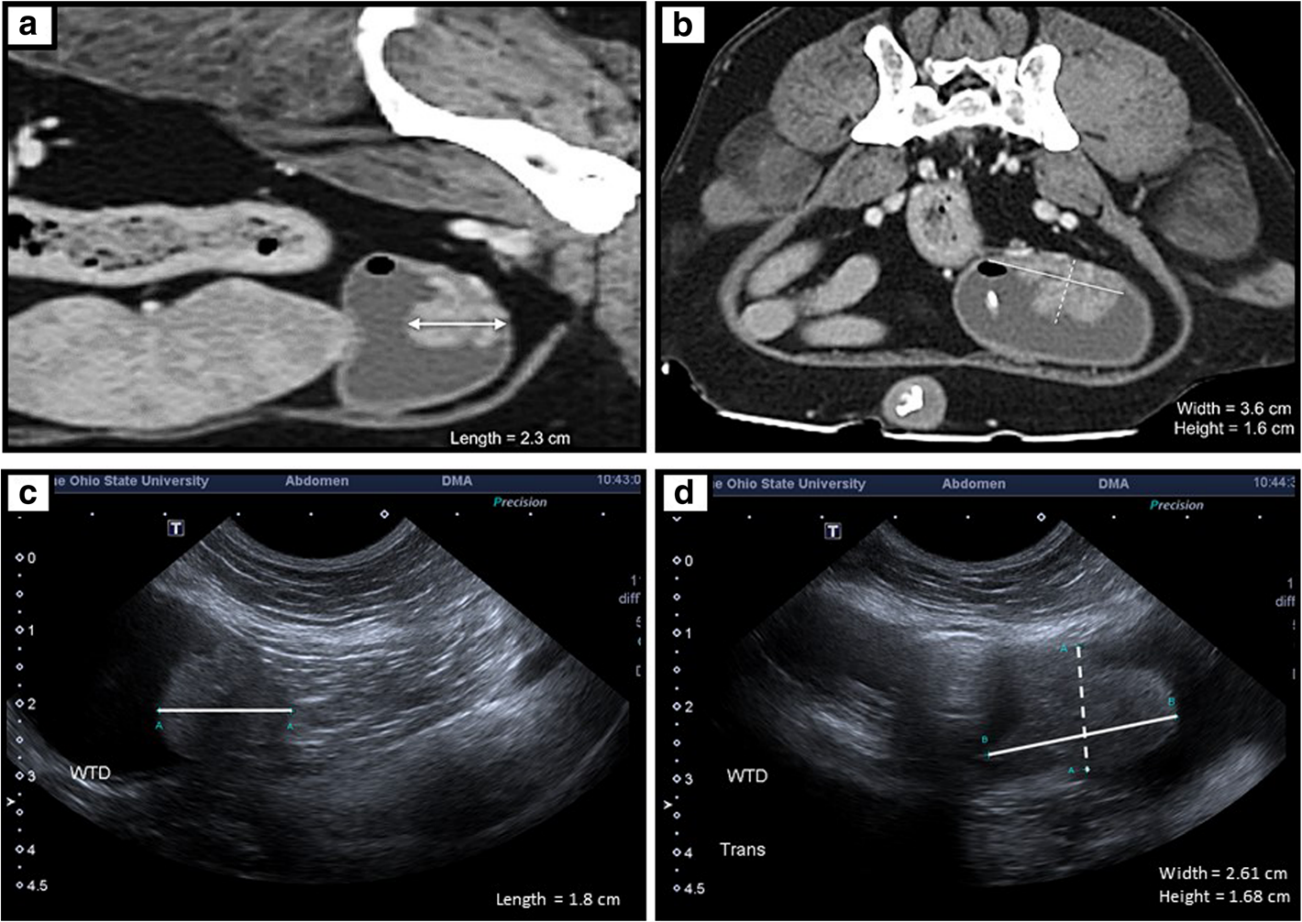
Tumor measurements as assessed by CT and Ultrasound. **a** CT sag 1 – Right parasagittal, post contrast enhanced CT image with the dog’s head to the left and tail to the right. An irregularly margined, soft tissue mass surrounded by less dense fluid (urine) is within the caudodorsal urinary bladder. The length of the mass is 2.3 cm (*white arrow*). **b** CT trans 1 – Transverse, post contrast enhanced CT images made through the caudal abdomen/caudal urinary bladder. An irregularly margined, soft tissue mass surrounded by less dense fluid (urine) is within the caudodorsal urinary bladder. The width of the mass (*solid white line*) is 3.6 cm; the height of the mass is 1.6 cm. A gas bubble is within the right dorsal aspect of the urinary bladder. A portion of the Foley catheter (*oblong white structure*) is ventral to the gas bubble. **c** US long – Parasagittal (long axis) ultrasound image of the urinary bladder. The dog’s head is to the left and the tail to the right. An irregularly margined mass is within the caudal urinary bladder. The length of the mass (*white line A*) is 1.8 cm. **d** US trans - Transverse (short axis) ultrasound image of the urinary bladder. An irregularly margined mass is within the caudal urinary bladder. The width of the mass (*white line B*) is 2.61 cm; the height of the mass (*dotted white line A*) is 1.68 cm

**Fig. 2:**
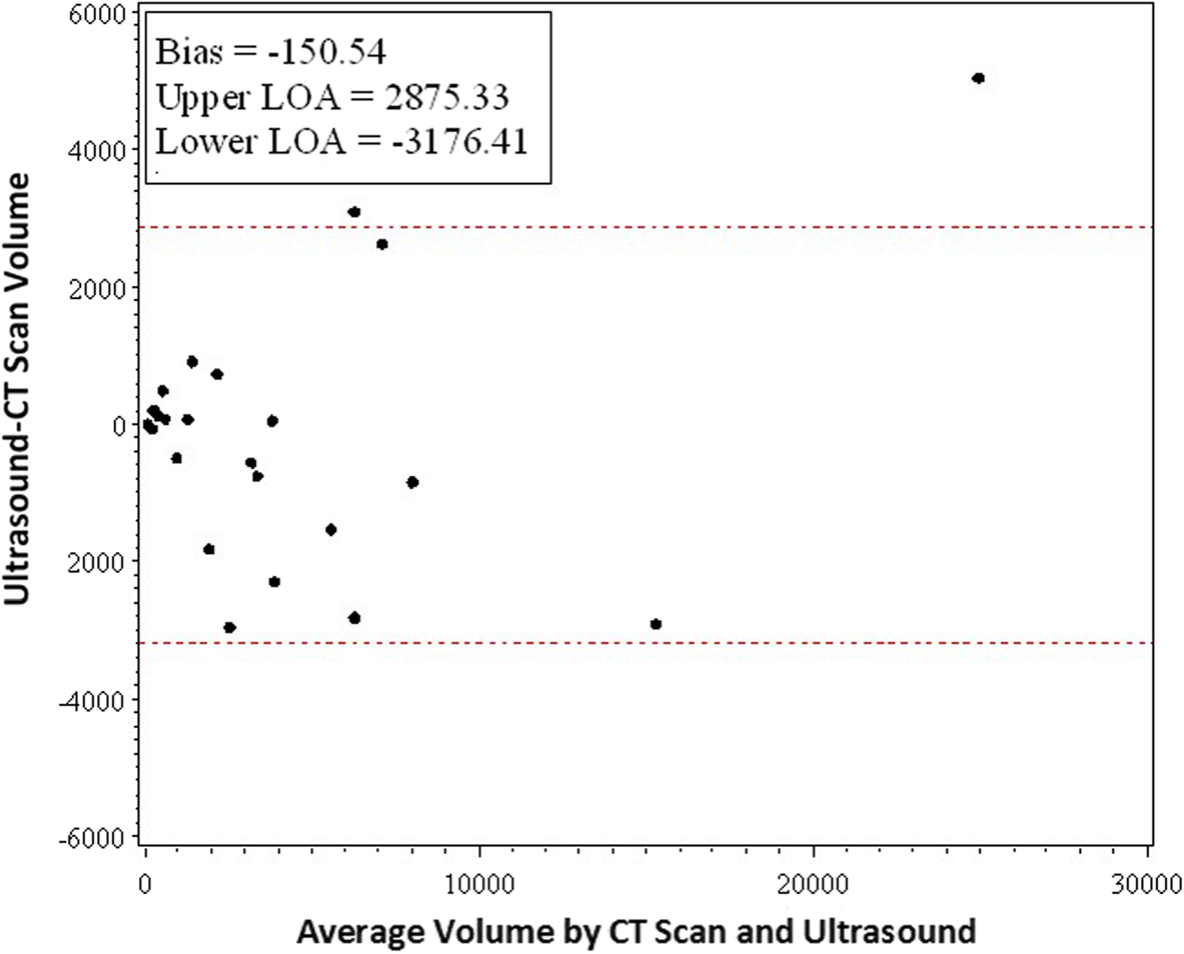
Bland-Altman plot of tumor volume for ultrasound versus CT. The disagreement plot shows the difference between ultrasound and CT methods against the average of the methods values for each subject. The two extreme lines are the +2 and −2 standard deviation

Four measurements (ultrasound assessments by two independent observers, CT measurements by two independent observers) were taken prior to treatment and at weeks 8 and 16 of the study (Fig. 1). As patients discontinued the study at various time points, nine out of ten dogs had data on the second visit, but only five dogs had values obtained for the week 16 assessment. Using the volumetric formula, the size of the tumor measured by ultrasound ranged from 65.48 to 28818.01 mm^3^ with a mean of 4113.96 mm^3^. The same tumors measured by CT scan ranged from 68.03 to 23625.36 mm^3^ with a mean of 4264.50 mm^3^. Repeated measures of tumor size (volume calculations) were tested for reliability within the respective modality (ultrasound vs. CT). Excellent reliability was achieved for both methods, while intra-class correlation (ICC) was slightly smaller with ultrasound (0.92, 95% CI: 0.82–0.96), compared to CT scan (0.98, 95% CI: 0.95–0.99). The values were then averaged across the two observers by each method at each visit. CT scan measurements were compared to ultrasound using overall concordance correlation coefficient (CCC) across all visits and CCC at baseline visit. Concordance between ultrasound and CT was demonstrated at the baseline visit (CCC: 0.96, 95% CI: 0.85–0.99) and across all visits (CCC: 0.92, 95% CI: 0.80–0.97). Concordance between CT and ultrasound was also assessed using Bland-Altman plots (Fig. 2).

**Fig. 3:**
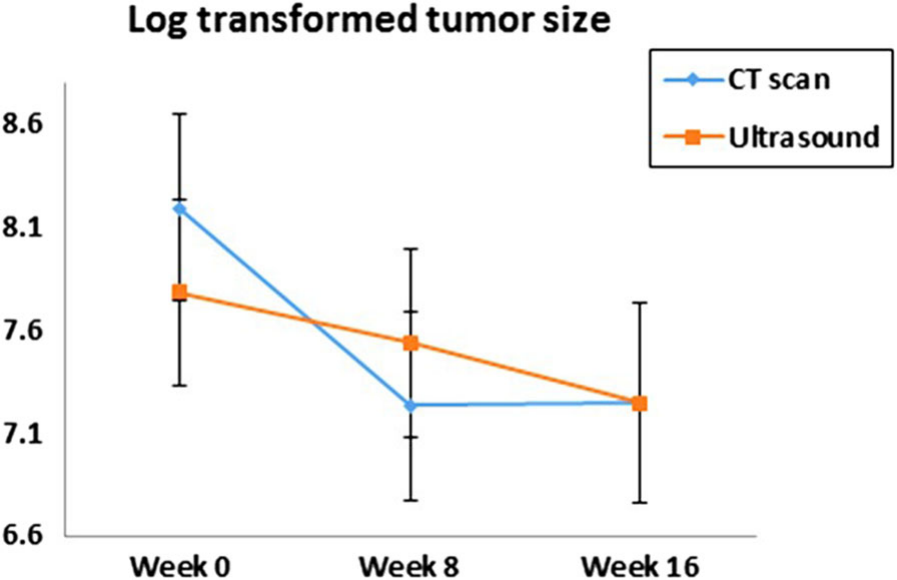
Change of tumor size across visits measured by CT scan and Ultrasound. Results shown are mean ± standard errors from mixed models including fixed effects of technician, method (ultrasound vs. CT), visit, and interactions of technician by visit, and method by visit

Results from the mixed effect model on change of tumor size across visits did not show significant difference between observers (*p* = 0.5 from test on interaction effect of observer by visit). However, measurements from CT scan indicated greater change in tumor size between week 0 and week 8, compared to measurements obtained by ultrasound (Fig. 3, *p* = 0.028).

### Response to therapy

The median duration of treatment was 108 days (arithmetic mean 87 days, range 14–112). Response to therapy at week 8 and week 12 was recorded based on a 30% reduction in tumor volume from week 0 (partial response, or PR), 20% increase in size from week 0 (progressive disease, or PD), or less than 30% reduction/less than 20% increase from week 0 (stable disease, or SD). Based on the mean AUS volume calculations, three patients completed the study with a biological response to treatment (2 PR, 1 SD). Based on the mean CT volume calculations, five patients completed the study with a biological response to treatment (2 PR, 3 SD). Three patients were withdrawn due to progressive clinical signs, 1 withdrew due to adverse events, and 1 withdrew due to owner non-compliance. The three patients with progression were withdrawn at week 8, week 12, or week 14. True progression free interval could not be calculated due to the 16-week duration of study.

Ultrasound measurements by individual assessors resulted in 3/9 or 5/9 dogs experiencing a PR at week 8, and 2/5 or 3/5 experiencing a PR at week 16. The inter-rater reliability was found to be Kappa = −0.24 with a 95% bootstrap confidence interval from −0.71 to 0.2. CT scan measurements by individual assessors resulted in 5/9 or 8/9 dogs experiencing a PR rate at week 8, and 2/5 or 3/5 experiencing a PR at week 16. The inter-rater reliability was found to be Kappa = 0.43 with a 95% bootstrap confidence interval from 0.14 to 0.84. The inter-rater reliability on clinical outcome (defined as tumor size reduction categorized by PR, SD, and PD) reached moderate agreement for CT scan, but poor agreement for ultrasound. Clinical outcomes based on averaged values from two operators were also recorded. Mean ultrasound assessment resulted in 4/9 dogs experiencing PR at week 8 and 2/5 dogs experiencing PR rate at week 16. By comparison, mean CT assessment resulted in 6/9 experiencing PR at week 8 and 2/5 experiencing PR at week 16. The Kappa statistic between ultrasound and CT scan was calculated as 0.16 with a 95% bootstrap confidence interval from −0.29 to 0.71, indicating slight agreement on tumor response between ultrasound and CT scan.

### Adverse events

Initial toceranib doses were adjusted to accommodate available tablet sizes (10, 15, and 50 mg) and later dose adjustments were made at clinician discretion due to adverse events (Table 1). The actual starting dose ranged from 2.27 to 2.74 mg/kg, with a mean dose of 2.59 mg/kg. Six patients received a toceranib dose reduction and 5 had a toceranib drug holiday during the study to due to clinical toxicities. Three of those patients required both a drug holiday and a dose reduction to remain on toceranib. One patient required a vinblastine dose reduction and 2 required vinblastine dose delays due to clinical toxicities.

**Table 1:** GI adverse events

GI adverse events	Grade	Patients	Total number events
Anorexia	1	9	13
	2	3	3
Weight Loss	1	6	7
Emesis	1	5	8
Nausea	1	2	2
Lethargy	1	4	5
Diarrhea	1	6	8
	2	3	4
Increased BUN, Normal Creatinine	1	2	3
	2	3	3

The most common adverse events were gastrointestinal (GI) in origin, and are documented in Table 1. All GI adverse events were grade 1 or 2. Nine dogs developed anorexia, six developed weight loss, five developed vomiting, two developed nausea, seven developed diarrhea, and five developed an increased BUN with normal creatinine, indicating possible GI bleeding. The second most commonly observed side effects were hematologic changes (Table 2), including grade 1 anemia (*n* = 5), grade 1 thrombocytopenia (*n* = 1), grade 1 or 2 neutropenia (*n* = 8), grade 3 neutropenia (*n* = 1), and grade 4 neutropenia (*n* = 1). Two patients with suspected pre-existing renal insufficiency (either a grade 1 BUN elevation or isosthenuria at screening) worsened while on study, developing either grade 1 or grade 2 elevations in creatinine. Both of these patients were on NSAIDs in addition to study medications, and it is unknown which medication exacerbated their conditions. Three patients developed grade 1 lameness or limb weakness, presumed to be secondary to muscle cramping, while on treatment. Three developed grade 2 or less elevation in liver values (ALP, AST, or ALT) during the study. One patient with a pre-existing grade 1 ALT elevation worsened while on study, developing a grade 4 ALT elevation. This patient was on both NSAIDs and toceranib and it is unknown which medication was responsible for worsening of the baseline values.

**Table 2:** Other adverse events

Other adverse events	Grade	Patients	Total number events
Increased ALT	1	1	1
	4	1	1
Increased AST	1	1	1
Increased ALP	2	1	1
Increased Creatinine	1	1	1
	2	1	1
Anemia	1	5	6
Thrombocytopenia	1	1	1
Neutropenia	1	6	7
	2	2	2
	3	1	1
	4	1	1
Limb Pain	1	3	3

## Discussion

Canine TCC remains a challenging disease to treat both surgically and medically, as most dogs have large invasive tumors, and response rates to chemotherapeutics are low (often <20%) and of short duration. Local disease resulting in substantial impairment of quality of life is the primary reason for euthanasia in the majority of canine TCC patients. Despite multiple efforts, there have been no significant improvements in outcome in the treatment of canine TCC over the past 15 years. A survival benefit is reported in human TCC with adjuvant chemotherapy (methotrexate, vinblastine, doxorubicin and cisplatin), with response rates of 35–65% in the setting of metastatic disease. However, recurrence rates are high with reported median survival times reaching 12–15 months in people with metastatic disease [43–45]. More recently, checkpoint inhibitor therapy through the use of monoclonal antibodies directed at PD1 have demonstrated single agent activity in people with metastatic TCC [46].

This clinical trial combined two therapies that have shown promise in treating canine TCC, vinblastine and toceranib, and evaluated the utility of ultrasound and CT for reproducibility and reliability in assessment of tumor volume and response to therapy. The underlying goal of this study was to provide preliminary data to inform subsequent prospective studies involving vinblastine/toceranib and imaging modalities in canine TCC. Our study found that response to therapy was not improved by using a combination of vinblastine and toceranib. In a previous study, single agent vinblastine was shown to induce stable disease in 50% of patients and partial responses in 36% of patients, with an overall biological response (PR and SD) rate of 86% [22]. Using this combination protocol, 30% of dogs experienced SD, and 40 or 60% (AUS or CT, respectively) of dogs experienced a PR to therapy at week 8 of the study. Therefore, the observed clinical benefit (PR and SD) reported herein is similar to published response rates using single-agent vinblastine [22]. It is possible that the current study did not demonstrate enhanced benefit of the vinblastine/toceranib combination as it required reduction in vinblastine dose to 1.6 mg/m^2^, representing a nearly 50% decrease from the 3 mg/m^2^ used for the single agent study. In addition, given the limited power of this study, it is possible that a larger population size would allow detection of a clinically significant difference with vinblastine/toceranib treatment compared to single-agent vinblastine.

Interestingly, tumor response at week 8 did not predict the duration of response to the combination protocol. Four patients withdrew prior to week 16 of the study (*n* = 3 PD, *n* = 1AE). Of the three patients that withdrew for PD, 2 had SD at the 8 week ultrasound evaluation, and all 3 had a biologic response (2 PR, 1 SD) at the 8 week CT. Only slight concordance was found between mean volume measurements obtained through CT and AUS. This indicates that the modalities are not interchangeable. Therefore, imaging comparisons should be undertaken using volume estimates obtained from the same imaging modality.

The repeatability of tumor volume measurement extrapolated from AUS using the equation (π/6 (L*W*H)) by different operators was excellent, however this technique was not shown to be useful for assessment of clinical patient status. Determining the response to treatment (PR, SD, or PD) for tumors based upon volumetric assessment obtained through AUS was found to have an unacceptably high variation between operators. Overall concordance in tumor response assessment (according to the categories of PR, SD, PR) was poor for ultrasound. Clinically significant differences (measurements resulting in a different response assessment) occurred 60% of the time. A total of seven patients had a clinically significant difference in operator measurements at least once while on trial.

Excellent inter-operator repeatability for tumor volume measurement using volumetric assessment extrapolated from CT with the equation (π/6 (L*W*H)). Determining the response to treatment (PR, SD, or PD) for tumors based upon volumetric assessment obtained through CT was found to have improved repeatability between operators when compared to ultrasound. Overall concordance in tumor response assessment (according to the categories of PR, SD, PR) was found to be moderate for CT. This resulted in clinically significant differences only 28% of the time. A total of three patients had clinically significant differences in their measurements at least once while on trial.

The proposed reason for the inter-operator variations in patient response assessment on both CT and ultrasound is a product of both the small size of the tumors evaluated and the volume equation utilized. While excellent concordance can be seen between volume assessments overall, small changes in numerical differences are magnified when the results are evaluated in terms of percent change from baseline. This compounds the existing inherent error in a volume calculation that assumes an ellipsoid shape for a tumor that may be highly irregular. The improved repeatability of response assessment by different operators with CT is notable, and indicates a more accurate estimate of changes in tumor volume is possible when the longest diameter measurements for length, width, and height are obtained through CT. Alternative methods for volume calculation, including the use of the perimeter method, may result in more accurate and repeatable volume assessments. This may permit improved tumor response assessment while still remaining simple and rapid enough for use within a standard clinical setting [32, 47].

Several inherent weaknesses in this study should be noted. The small sample size in this pilot study limits robust interpretation of the results and increases the risk of making a type II error. In addition, this study lacked a prospective control group and was not randomized, making direct comparisons difficult.

As mentioned previously, volumetric tumor assessment by either modality at 8 weeks did not correlate with duration on study, indicating that neither AUS nor CT imaging will accurately predict the duration of response to therapy. Based on inter-operator variability and the lack of correlation between clinical outcome and imaging, clinical decision-making based upon image findings alone is not recommended. Alternative measures of disease burden, including activating BRAF mutations, survivin and telomerase activity have also been evaluated in TCC [48–51].

## Conclusions

The data presented in this pilot study indicate that the combination of toceranib and vinblastine treatment in canine TCC provide no obvious improvement in clinical benefit (PR and SD) when compared to previously studied treatment modalities. In the present study, the most consistent assessment of tumor response to treatment was obtained using tumor volume extrapolated from CT. Given the variability in assessment of tumor response between different operators and the poor correlation between measurements and clinical outcome, making clinical decisions based upon imaging characteristics alone is not recommended, however additional prospective studies are warranted to confirm these findings.

## Abbreviations

AEs: Adverse events
ALP: Alkaline phosphatase
ALT: Alanine aminotransferase
AST: Aspartate aminotransferase
AUS: Abdominal ultrasound
BUN: Blood urine nitrogen
CBC: Complete blood count
CCC: Concordance correlation coefficient
COX1/COX2: Cyclooxygenase 1 and 2
CT: Computerized tomography
DICOM: Digital imaging and communication in medicine
ECOG: Eastern cooperative oncology group
GI: Gastrointestinal
IACUC: Institutional Animal Care and Use Committee
ICC: Intra-class correlation
KIT: CD 117
MRI: Magnetic resonance imaging
M-VAC: Methotrexate, vinblastine, doxorubicin and cisplatin
NSAID: Non-steroidal anti-inflammatory
PD: Progressive disease
PD1: Programed cell death ligand 1
PDGFR: Platelet derived growth factor
PR: Partial response
RTK: Receptor tyrosine kinase
SD: Stable disease
TCC: Transitional cell carcinoma
ULN: Upper limit of normal
UPC: Urine protein creatinine ratio
VCOG-CTCAE: Veterinary cooperative oncology group common terminology criteria for adverse events
VEGFR: Vascular endothelial growth factor

## References

[1] Fulkerson CM, Knapp DW (2015). Management of transitional cell carcinoma of the urinary bladder in dogs: a review. Vet J.

[2] Knapp DW, Glickman NW, DeNicola DB, Bonney PL, Lin TL, Glickman LT (2000). Naturally-occuring canine transitional cell carcinoma of the urinary bladder: A relevant model of human invasive bladder cancer. Urol Oncol.

[3] Knapp DW, Ramos-Vara JA, Moore GE, Dhawan D, Bonney PL, Young KE (2014). Urinary bladder cancer in dogs, a naturally occurring model for cancer biology and drug development. ILAR J.

[4] Mutsaers AJ, Widmer WR, Knapp DW (2003). Canine transitional cell carcinoma. J Vet Intern Med.

[5] Hayes HM (1976). Canine bladder cancer: epidemiologic features. Am J Epidemiol.

[6] Norris AM, Laing EJ, Valli VE, Withrow SJ, Macy DW, Ogilvie GK, Tomlinson J, McCaw D, Pidgeon G, Jacobs RM (1992). Canine bladder and urethral tumors: a retrospective study of 115 cases (1980–1985). J Vet Intern Med.

[7] Glickman LT, Raghavan M, Knapp DW, Bonney PL, Dawson MH (2004). Herbicide exposure and the risk of transitional cell carcinoma of the urinary bladder in Scottish Terriers. J Am Vet Med Assoc.

[8] Glickman LT, Schofer FS, McKee LJ, Reif JS, Goldschmidt MH (1989). Epidemiologic study of insecticide exposures, obesity, and risk of bladder cancer in household dogs. J Toxicol Environ Health.

[9] Boston S, Singh A (2014). Total cystectomy for treatment of transitional cell carcinoma of the urethra and bladder trigone in a dog. Vet Surg.

[10] Stone EA, George TF, Gilson SD, Page RL (1996). Partial cystectomy for urinary bladder neoplasia: surgical technique and outcome in 11 dogs. J Small Anim Pract.

[11] Stone EA, Withrow SJ, Page RL, Schwarz PD, Wheeler SL, Seim HB (1988). Ureterocolonic anastomosis in ten dogs with transitional cell carcinoma. Vet Surg.

[12] Blackburn AL, Berent AC, Weisse CW, Brown DC (2013). Evaluation of outcome following urethral stent placement for the treatment of obstructive carcinoma of the urethra in dogs: 42 cases (2004–2008). J Am Vet Med Assoc.

[13] McMillan SK, Knapp DW, Ramos-Vara JA, Bonney PL, Adams LG (2012). Outcome of urethral stent placement for management of urethral obstruction secondary to transitional cell carcinoma in dogs: 19 cases (2007–2010). J Am Vet Med Assoc.

[14] Weisse C, Berent A, Todd K, Clifford C, Solomon J (2006). Evaluation of palliative stenting for management of malignant urethral obstructions in dogs. J Am Vet Med Assoc.

[15] Liptak JM, Brutscher SP, Monnet E, Dernell WS, Twedt DC, Kazmierski KJ, Walter CU, Mullins MN, Larue SM, Withrow SJ (2004). Transurethral resection in the management of urethral and prostatic neoplasia in 6 dogs. Vet Surg.

[16] Mohammed SI, Bennett PF, Craig BA, Glickman NW, Mutsaers AJ, Snyder PW, Widmer WR, DeGortari AE, Bonney PL, Knapp DW (2002). Effects of the cyclooxygenase inhibitor, piroxicam, on tumor response, apoptosis, and angiogenesis in a canine model of human invasive urinary bladder cancer. Cancer Res.

[17] Knapp DW, Richardson RC, Chan TC, Bottoms GD, Widmer WR, DeNicola DB, Teclaw R, Bonney PL, Kuczek T (1994). Piroxicam therapy in 34 dogs with transitional cell carcinoma of the urinary bladder. J Vet Intern Med.

[18] Boria PA, Glickman NW, Schmidt BR, Widmer WR, Mutsaers AJ, Adams LG, Snyder PW, DiBernardi L, de Gortari AE, Bonney PL (2005). Carboplatin and piroxicam therapy in 31 dogs with transitional cell carcinoma of the urinary bladder. Vet Comp Oncol.

[19] Knapp DW, Glickman NW, Widmer WR, DeNicola DB, Adams LG, Kuczek T, Bonney PL, DeGortari AE, Han C, Glickman LT (2000). Cisplatin versus cisplatin combined with piroxicam in a canine model of human invasive urinary bladder cancer. Cancer Chemother Pharmacol.

[20] Henry CJ, McCaw DL, Turnquist SE, Tyler JW, Bravo L, Sheafor S, Straw RC, Dernell WS, Madewell BR, Jorgensen L (2003). Clinical evaluation of mitoxantrone and piroxicam in a canine model of human invasive urinary bladder carcinoma. Clin Cancer Res.

[21] Allstadt SD, Rodriguez CO, Boostrom B, Rebhun RB, Skorupski KA (2015). Randomized phase III trial of piroxicam in combination with mitoxantrone or carboplatin for first-line treatment of urogenital tract transitional cell carcinoma in dogs. J Vet Intern Med.

[22] Arnold EJ, Childress MO, Fourez LM, Tan KM, Stewart JC, Bonney PL, Knapp DW (2011). Clinical trial of vinblastine in dogs with transitional cell carcinoma of the urinary bladder. J Vet Intern Med.

[23] Schrempp DR, Childress MO, Stewart JC, Leach TN, Tan KM, Abbo AH, de Gortari AE, Bonney PL, Knapp DW (2013). Metronomic administration of chlorambucil for treatment of dogs with urinary bladder transitional cell carcinoma. J Am Vet Med Assoc.

[24] Withrow SJ, Gillette EL, Hoopes PJ, McChesney SL (1989). Intraoperative irradiation of 16 spontaneously occurring canine neoplasms. Vet Surg.

[25] Poirier VJ, Forrest LJ, Adams WM, Vail DM (2004). Piroxicam, mitoxantrone, and coarse fraction radiotherapy for the treatment of transitional cell carcinoma of the bladder in 10 dogs: a pilot study. J Am Anim Hosp Assoc.

[26] Nolan MW, Kogan L, Griffin LR, Custis JT, Harmon JF, Biller BJ, Larue SM (2012). Intensity-modulated and image-guided radiation therapy for treatment of genitourinary carcinomas in dogs. J Vet Intern Med.

[27] Choy K, Fidel J (2016). Tolerability and Tumor Response of a Novel Low-Dose Palliative Radiation Therapy Protocol in Dogs with Transitional Cell Carcinoma of the Bladder and Urethra. Vet Radiol Ultrasound.

[28] Parfitt SL, Milner RJ, Salute ME, Hintenlang DE, Farese JP, Bacon NJ, Bova FJ, Rajon DA, Lurie DM (2011). Radiosensitivity and capacity for radiation-induced sublethal damage repair of canine transitional cell carcinoma (TCC) cell lines. Vet Comp Oncol.

[29] Nolan MW, Gieger TL, Vaden SL (2015). Management of transitional cell carcinoma of the urinary bladder in dogs: important challenges to consider. Vet J.

[30] Browne RF, Meehan CP, Colville J, Power R, Torreggiani WC (2005). Transitional cell carcinoma of the upper urinary tract: spectrum of imaging findings. Radiographics.

[31] Hume C, Seiler G, Porat-Mosenco Y, Caceres A, Shofer F, Sorenmo K (2010). Cystosonographic measurements of canine bladder tumours. Vet Comp Oncol.

[32] Naughton JF, Widmer WR, Constable PD, Knapp DW (2012). Accuracy of three-dimensional and two-dimensional ultrasonography for measurement of tumor volume in dogs with transitional cell carcinoma of the urinary bladder. Am J Vet Res.

[33] Eisenhauer EA, Therasse P, Bogaerts J, Schwartz LH, Sargent D, Ford R, Dancey J, Arbuck S, Gwyther S, Mooney M (2009). New response evaluation criteria in solid tumours: revised RECIST guideline (version 1.1). Eur J Cancer.

[34] London CA, Hannah AL, Zadovoskaya R, Chien MB, Kollias-Baker C, Rosenberg M, Downing S, Post G, Boucher J, Shenoy N (2003). Phase I dose-escalating study of SU11654, a small molecule receptor tyrosine kinase inhibitor, in dogs with spontaneous malignancies. Clin Cancer Res.

[35] Arantes-Rodrigues R, Pinto-Leite R, Fidalgo-Goncalves L, Palmeira C, Santos L, Colaco A, Oliveira P (2013). Synergistic effect between cisplatin and sunitinib malate on human urinary bladder-cancer cell lines. Biomed Res Int.

[36] Wu CL, Ping SY, Yu CP, Yu DS (2012). Tyrosine kinase receptor inhibitor-targeted combined chemotherapy for metastatic bladder cancer. Kaohsiung J Med Sci.

[37] Robat C, London C, Bunting L, McCartan L, Stingle N, Selting K, Kurzman I, Vail DM (2012). Safety evaluation of combination vinblastine and toceranib phosphate (Palladia(R)) in dogs: a phase I dose-finding study. Vet Comp Oncol.

[38] Veterinary cooperative oncology group - common terminology criteria for adverse events (VCOG-CTCAE) following chemotherapy or biological antineoplastic therapy in dogs and cats v1.1. Vet Comp Oncol 2011. doi: 10.1111/j.1476-5829.2011.00283.x 10.1111/vco.283 28530307

[39] Somville J, De Beuckeleer L, De Schepper A, Verstreken J, Taminiau A (2001). Reliability of measuring volume by different methods for tumors of the musculoskeletal system. Acta Orthop Belg.

[40] Bland JM, Altman DG (1986). Statistical methods for assessing agreement between two methods of clinical measurement. Lancet.

[41] Carrasco JL, Phillips BR, Puig-Martinez J, King TS, Chinchilli VM (2013). Estimation of the concordance correlation coefficient for repeated measures using SAS and R. Comput Methods Programs Biomed.

[42] Kenward MG, Roger JH (1997). Small sample inference for fixed effects from restricted maximum likelihood. Biometrics.

[43] Oosterlinck W, Lobel B, Jackse G, Malmstrom PU, Stockle M, Strenberg C, European Association of U (2002). [EAU Recommendations 2001. “Guidelines on bladder cancer”]. Prog Urol.

[44] Oosterlinck W, Lobel B, Jakse G, Malmstrom PU, Stockle M, Sternberg C, European Association of Urology Working Group on Oncological U (2002). Guidelines on bladder cancer. Eur Urol.

[45] Vaishampayan U (2009). Systemic therapy of advanced urothelial cancer. Curr Treat Options Oncol.

[46] Aoun F, Kourie HR, Sideris S, Roumeguere T, Velthoven R, Gil T (2015). Checkpoint inhibitors in bladder and renal cancers: results and perspectives. Immunotherapy.

[47] Sorensen AG, Patel S, Harmath C, Bridges S, Synnott J, Sievers A, Yoon YH, Lee EJ, Yang MC, Lewis RF (2001). Comparison of diameter and perimeter methods for tumor volume calculation. J Clin Oncol.

[48] Decker B, Parker HG, Dhawan D, Kwon EM, Karlins E, Davis BW, Ramos-Vara JA, Bonney PL, McNiel EA, Knapp DW (2015). Homologous Mutation to Human BRAF V600E Is Common in Naturally Occurring Canine Bladder Cancer--Evidence for a Relevant Model System and Urine-Based Diagnostic Test. Mol Cancer Res.

[49] Ku JH, Godoy G, Amiel GE, Lerner SP (2012). Urine survivin as a diagnostic biomarker for bladder cancer: a systematic review. BJU Int.

[50] McCleary-Wheeler AL, Williams LE, Hess PR, Suter SE (2010). Evaluation of an in vitro telomeric repeat amplification protocol assay to detect telomerase activity in canine urine. Am J Vet Res.

[51] Mochizuki H, Shapiro SG, Breen M (2015). Detection of BRAF Mutation in Urine DNA as a Molecular Diagnostic for Canine Urothelial and Prostatic Carcinoma. PLoS One.

