# Erratum to: A pilot study of toceranib/vinblastine therapy for canine transitional cell carcinoma

**DOI:** 10.1186/s12917-016-0934-y

**Published:** 2016-12-30

**Authors:** Sarah B. Rippy, Heather L. Gardner, Sandra M. Nguyen, Emma E. Warry, Roberta F. Portela, William Tod Drost, Eric T. Hostnik, Eric M. Green, Dennis J. Chew, Juan Peng, Cheryl A. London

**Affiliations:** 1 University of Missouri, Columbia, MO USA; 2 Departments of Veterinary Clinical and Biosciences, The Ohio State University, 454 Veterinary Medical Academic Building, 1925 Coffey Rd., Columbus, OH 43210 USA; 3 Premier Veterinary Group of Chicago, Chicago, IL USA; 4 Department of Biostatistics, The Ohio State University, Columbus, OH USA

## Erratum

The original article [1] contained an error whereby the co-author, Roberta F. Portela’s name was misspelt using an incorrect letter for the middle initial.

This error has now been corrected in the original article.
